# Al_13_@Pt_42_ Core-Shell Cluster for Oxygen Reduction Reaction

**DOI:** 10.1038/srep05205

**Published:** 2014-06-06

**Authors:** B. B. Xiao, Y. F. Zhu, X. Y. Lang, Z. Wen, Q. Jiang

**Affiliations:** 1 Key Laboratory of Automobile Materials, Ministry of Education, and School of Materials Science and Engineering, Jilin University, Changchun 130022, China

## Abstract

To increase Pt utilization for oxygen reduction reaction (ORR) in fuel cells, reducing particle sizes of Pt is a valid way. However, poisoning or surface oxidation limits the smallest size of Pt particles at 2.6 nm with a low utility of 20%. Here, using density functional theory calculations, we develop a core-shell Al_13_@Pt_42_ cluster as a catalyst for ORR. Benefit from alloying with Al in this cluster, the covalent Pt-Al bonding effectively activates the Pt atoms at the edge sites, enabling its high utility up to 70%. Valuably, the adsorption energy of O is located at the optimal range with 0.0–0.4 eV weaker than Pt(111), while OH-poisoning does not observed. Moreover, ORR comes from O_2_ dissociation mechanism where the rate-limiting step is located at OH formation from O and H with a barrier of 0.59 eV, comparable with 0.50 eV of OH formation from O and H_2_O on Pt(111).

Proton exchange membrane fuel cells (PEMFCs) are promising candidates for mobile and transport applications due to their high energy density, zero emissions, relatively low operating temperature, and minimal corrosion problems^1^. Pt nanoparticles supported on carbon are commonly used as catalysts of the cathode for the oxygen reduction reaction (ORR)^2^. It is observed that the ORR activity highly depends on the size of nanoparticles, where Pt nanoparticles with diameters (*D*) of 2–5 nm are regarded as the best^3^,^4^. This is because the percentage of atoms on the active Pt(111) facets over the total number of atoms *n*, denoting as *R*_(111)_, reaches the maximum^2^,^5^,^6^. If the particle takes an (111)-enclosed icosahedral shape as shown in Figure 1, in order to maximize *R*_(111)_, the corresponding *D* (*D*_c_) is 2.6 nm with *n* = 561, which brings out *R*_(111)_ = 20%. Further reducing *D* could increase the surface/volume ratio, and the percentages of edge and vertex sites (*R*_e_ and *R*_v_) on the particle surfaces unfortunately increase where *R*_e_ becomes predominant below *D*_c_. Those Pt atoms at the low-coordinated sites are adverse for ORR due to the strong binding of O-containing intermediates^4^,^7^,^8^,^9^,^10^. Thus, activating edge and vertex sites is the main challenge to miniaturize *D*.

As adsorption energies of all intermediates of ORR are related to the O adsorption energy [*E*_ads_(O)] on (111) surfaces of transition metals, the activity is proposed to be a function of *E*_ads_(O)^2^,^11^,^12^,^13^. It has demonstrated that *E*_ads_(O) of a catalyst with the best activity for ORR should be 0.0–0.4 eV weaker than that of Pt(111)^2^,^9^,^13^. Furthermore, a volcano activity curve based on the adsorption energy of OH [*E*_ads_(OH)] is present where *E*_ads_(OH) is 0.0–0.2 eV weaker than that of Pt(111), due to the scaling relationship between *E*_ads_(O) and *E*_ads_(OH)^2^,^6^,^12^,^14^. Note that the coordination numbers of the vertex atoms and the edge atoms of nanoparticles differ from that of (111) surface of nine. Thus, the scaling relationship between *E*_ads_(O) and *E*_ads_(OH) may be changed and *E*_ads_(O) and *E*_ads_(OH) must be both inquired separately.

On the other hand, numerous experimental and theoretical studies have been carried out to study the kinetics of ORR mechanisms^2^,^15^,^16^,^17^,^18^. To our knowledge, there are mainly three mechanisms: O_2_ dissociation, OOH dissociation and H_2_O_2_ dissociation. Qi *et al.* have demonstrated that in the gas phase, instead of the high barrier (*E*_a_) of OH formation from O and H with a value of 0.91 eV, OH is originated from OOH and H with *E*_a_ = 0.31 eV^15^,^16^. However, this path is absent because H_2_O_2_ formation is forbidden in solution^17^. Recently, a new path is proposed: OH formation in solution comes from O and H_2_O, and ORR on Pt(111) is essentially carried out by O_2_ dissociation mechanism, namely O_2_ dissociation (*E*_a_ = 0.00 eV), OH formation (*E*_a_ = 0.50 eV) and H_2_O formation (*E*_a_ = 0.24 eV)^18^. It is doubted that whether ORR mechanism is changed due to the presence of the low-coordinated atoms at nano size. Thus, in order to fully understand the catalysis, the kinetics of ORR mechanisms needs to be further explored.

Alloying is a general technique to improve the ORR activity and stability of catalysts^13^,^14^,^19^,^20^,^21^. Present works have been mainly concentrated on Pt-based alloys consisting of Pt and the late TM elements in the 3*d* series, typically Pt_3_Fe, Pt_3_Co and Pt_3_Ni. The alloys show better catalysis activity than Pt alone^22^. However, the severe degradation of catalysis and stability of these alloys during the voltage cycling in acids as a consequence of the continuous dissolution of TM atoms are present^22^,^23^. This can be understood by their negligible heat of formation^13^,^24^. Following the suggestion of Greeley et al., strong binding between Pt and other alloying elements is needed to improve the stability of any new alloy systems^13^. Since the formation energy of Pt_3_Al is much more negative than Pt-based alloys with late TM elements^24^, Pt_3_Al should be a good substitute of the above Pt-based alloys. It must be admitted that Pt-based alloys with early TM elements show good activity and stability, such as Pt_3_Sc and Pt_3_Y^13^,^22^. However, these works are still located at the transition metals and the activity enhancement is resulted from the *d-d* interaction to modify the *d* band of Pt surface atoms^13^,^22^. Thus, from the electronic aspect, the *p-d* interaction of Al-Pt systems would provide an attempt to look beyond the Pt-TM systems and explore novel catalysts.

Recent DFT calculations show that a core-shell structure plays an important role in increasing the stability of Pt-based nanoalloys, such as Co_13_@Pt_42_ and Rh_13_@Pt_42_^25^,^26^ where Pt shell benefits for the stability of the catalysts under the electrochemical environment^21^,^23^,^27^,^28^,^29^. Thus, we here develop a new core-shell Al_13_@Pt_42_ cluster as ORR catalyst, whose surface is assembled with the twelve vertex atoms Pt_v_ and the thirty edge atoms Pt_e_. In addition, Al_13_ cluster with icosahedral symmetry have been shown to exhibit enhanced stability compared with other isomers^30^,^31^. What is more, the ligand stabilized Al nanoparticles with the size range of 1.5 and 4 nm have been synthesized^32^,^33^. It is noteworthy that although Al@Pt core-shell nanoparticles have not been synthesized to date, Al@Cu and Al@Co ones are fabricated via a displacement reaction^34^,^35^. Although these particles had large particle sizes about 5 μm, the utilized experimental technique could also be applied to fabricate Al@Pt core-shell nanostructures as long as the size of Al nanoparticles is small, which has been be synthesized without difficulties^32^,^33^. Note that although Al could be easily oxidized it has been easily avoided by an inert atmosphere^33^. At last, it is emphasized that our work offers only a theoretical prediction and we hope this new Al@Pt cluster will be picked up by experimentalists for empirical verification.

In light of our calculation by using Density Functional Theory (DFT), Al@Pt cluster possesses good stability due to the covalent bonding between Al_13_ core and Pt_42_ shell. Also, *E*_ads_(O) is located at the optimal range while *E*_ads_(OH) on Pt_e_ is 0.30 eV weaker than Pt(111). Furthermore, rate-limiting step (RDS) of the ORR reaction though O_2_ dissociation mechanism is located at OH formation from O and H (*E*_a_ = 0.59 eV). This barrier is comparable with 0.50 eV of Pt(111)^18^. Thus, alloying with Al effectively activates Pt_e_ atoms and lets the utility of Pt reach 70% (30 edge Pt_e_ atoms from the total 42 Pt atoms).

## Results

**Figure 2:**
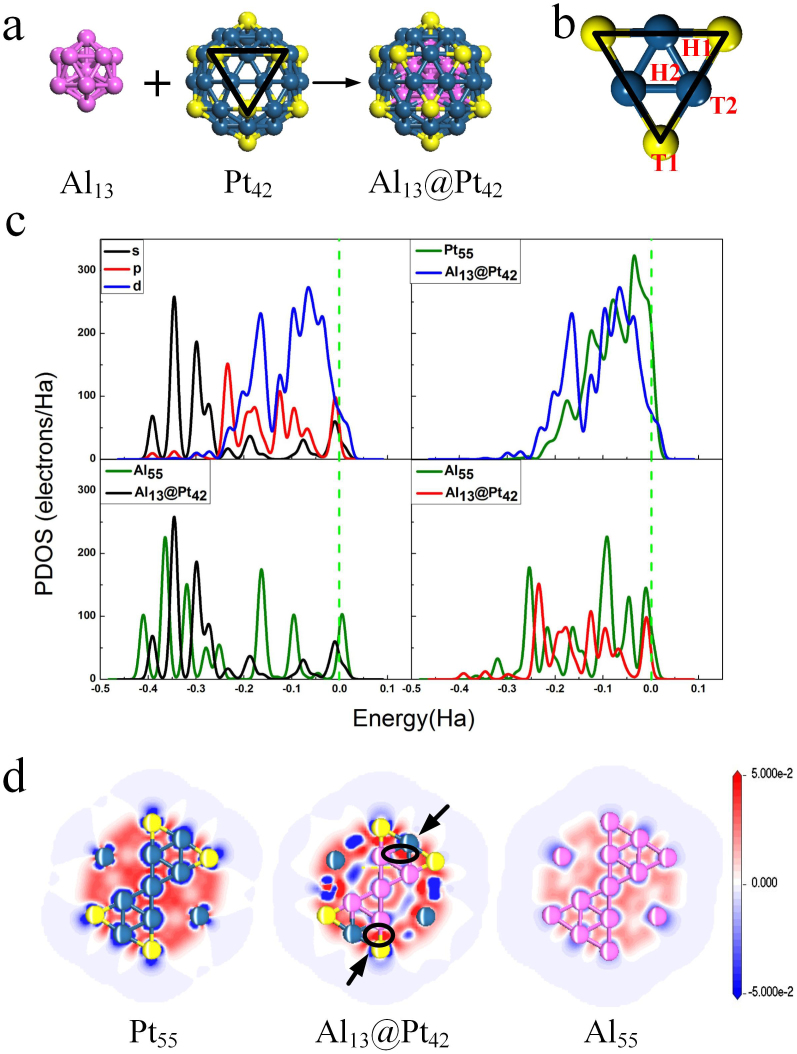
(a) The structure of icosahedral Al_13_@Pt_42_ cluster. Atoms in yellow denotes vertex Pt_v_, edge Pt_e_ atoms are in blue. Purple shows Al atoms. (b) The adsorption sites of high-symmetry on a triangular face. T1 and T2 top sites are located atop of Pt_v_ and Pt_e_, respectively; H1 and H2 show hcp and fcc hollow sites respectively. The former is surrounded by Pt_v_ and Pt_e_ while the latter is surrounded by only Pt_e_. (c) The partial density of states (PDOS). Top left is PDOS of Al_13_@Pt_42_. For clarity, the intensity of *d*-electrons is reduced to the one tenth. Top right is *d*-electron PDOS of Pt_42_ shell on Pt_55_ and Al_13_@Pt_42_. Bottom left and right are *s*- and *p*-electron PDOS of Al_13_ core in Al_55_ and Al_13_@Pt_42_. (d) The plot of electron density difference Δρ.The loss and enrichment of electrons are indicated in blue and red. Here, Al_55_ denotes 55-atomic Al cluster with icosahedral shape.

Figure 2(a) shows a core-shell Al_13_@Pt_42_ cluster (*n* = 55) with an icosahedral structure where 13 Al atoms form an icosahedral core and all Pt atoms are located on the shell. All Pt atoms are all low-coordinated, which consist of 6-coordination vertex atoms (Pt_v_) and 8-coordination edge atoms (Pt_e_). The Al_13_@Pt_42_ possesses a high symmetry and stability (the mean binding energy *E*_b_ = −4.8 eV/atom compared with −4.75 eV/atom of Pt_55_ according to our calculation). To understand physically the interaction between Al core and Pt shell of Al_13_@Pt_42_, partial density of states (PDOS) is shown in Figure 2(c). Compared with Pt_55_, the *d* band of Pt_42_ shell on Al_13_@Pt_42_ is moved away from the Fermi energy *E*_F_. That is, the *d* band center changes from −1.99 of Pt_55_ to −2.54 eV of Al_13_@Pt_42_. Furthermore, the *d* band of alloy cluster is clearly more discrete. The *d* band of Pt_42_ shell is concentrated in between 0 to −6.8 eV. For Al_13_ core, the *p* band has the same trend with the *d* band of Pt_42_ shell, which denotes the strong orbital hybridization. It is obvious that the *d* orbitals (at −1.7, −2.6, −3.4, −4.5, −5.5 and −6.3 eV) interact with the *p* orbitals (at −1.8, 2.6, −3.4, −4.9 and −6.3 eV) and weak *p-d* hybridization is present at −8.1 eV below *E*_F_. On the other hand, the main of *s* band is located below −6.8 eV. Compared with *p-d* hybridization, the *s-d* interaction is weak, appearing at −5.1, −6.4, −7.4 and −8.1 eV below *E*_F_. Therefore, the enhancement in stability is dominated by hybridization between Pt-5*d* band and Al-3*p* band. To confirm this interaction, the electron density difference Δρ calculated is presented in Figure 2(d). Obviously, electrons are accumulated between Pt and Al atoms, which are compatible with the observation of the corresponding PDOS and demonstrates the partial formation of the covalent Pt-Al bonds.

In order to further confirm the stability of Al_13_@Pt_42_, we consider the stability of Fe_13_@Pt_42_, Co_13_@Pt_42_ and Ni_13_@Pt_42_ for a comparison purpose as the three alloying elements have been well studied^36^,^37^,^38^. Table 1 lists the core-shell interaction energy *E*_cs_, which could interpret enhanced phenomenon^13^,^26^,^39^, and the dissolution potential *U*_diss_(M_13_@Pt_42_) of the Pt_42_ shell in M_13_@Pt_42_ icosahedral clusters (M = Al, Fe, Co, Ni). It is found that due to the alloying, *U*_diss_(M_13_@Pt_42_) of clusters are enhanced compared with that of Pt_55_. That is, the stronger *E*_cs_ makes the higher dissolution resistance, which is similar to the relationship between the alloy formation energy and the ORR stability of the Pt_3_M bulk^13^,^39^. The corresponding order is Al_13_@Pt_42_ > Fe_13_@Pt_42_ > Co_13_@Pt_42_ > Ni_13_@Pt_42_ > Pt_55_. Thus, we expect that the stability of Al_13_@Pt_42_ acted as ORR catalysts is well.

*E*_ads_(O) and *E*_ads_(OH) on Al_13_@Pt_42_ are firstly examined. For comparison purpose, these values on Pt_55_ and Pt(111) are also calculated. According to previous studies, we focused on the adsorption of O on hollow sites and OH on atop sites as the adsorption sites shown in Figure 2(b)^13^,^40^. The corresponding *E*_ads_(O) and *E*_ads_(OH) values are listed in Table 2. The most favored *E*_ads_(O) and *E*_ads_(OH) values on Pt(111) are −4.51 on fcc site and −2.45 eV on atop site [the reported results are *E*_ads_(O) = −4.21 eV and *E*_ads_(OH) = −2.31 eV, respectively, being in accord with our data^41^,^42^]. For O adsorption on Pt_55_, *E*_ads_(O) are −4.56 and −4.71 eV on H1 and H2 sites, respectively, which are stronger than that of Pt(111). Similarly, compared with Pt(111), OH adsorption are stronger with *E*_ads_(OH) of −3.13 eV on T1 site and −2.91 eV on T2 site. Therefore, Pt_55_ are both O and OH poisoned due to the enhanced adsorption ability of the low-coordinated Pt atoms^43^. On the other hand, for Al_13_@Pt_42_, *E*_ads_(O) are −4.15 and −4.25 eV on H1 and H2 sites, respectively, which are 0.36 and 0.26 eV weaker than that of Pt(111). Therein, the *E*_ads_(O) values are located at the optimal range for ORR^2^,^13^. For OH adsorption on T1 site, *E*_ads_(OH) is −2.81 eV and is 0.36 eV stronger compared with Pt(111). However, as OH on T2 site, *E*_ads_(OH) is 0.30 eV weaker than that of Pt(111) with a value of −2.15 eV. The scaling relationship between O and OH is broken on Al_13_@Pt_42_^11^,^12^. Although there is serious OH-poisoning at T1 site, OH-poisoning at T2 site is absent. Thus, OH adsorption on T2 site can easily be removed, and the recovery of T2 site for the next ORR cycle could take place. In light of viewpoint of OH-poisoning, it is likely that the only edge atoms (Pt_e_) of Al_13_@Pt_42_ are effective for ORR.

**Figure 3:**
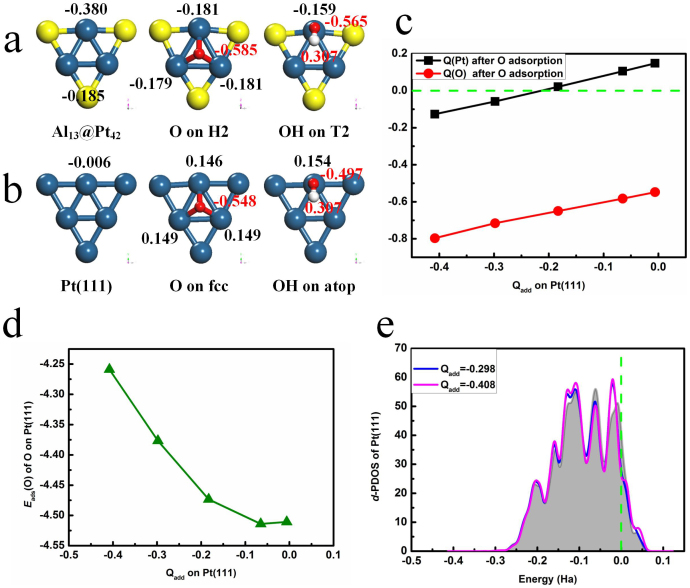
The Mulliken charges Q of Al_13_@Pt_42_ (a) and Pt (111) (b). (c) The Mulliken charges Q on Pt(111) with different Q_add_. (d) The *E*_ads_(O) on Pt(111) with different Q_add_. (e) The plot of *d*-electron PDOS of Pt(111) with different Q_add_. For comparison, *d*-electron PDOS of Pt(111) without Q_add_ is shown in shaded. A negative value means electronic gain and a positive value means electronic loss.

The above results are supported by the relationship between electronic structures and atomic ones of Al_13_@Pt_42_. It is known that surface atoms with larger coordination number have a lower *d* band center and weaker adsorption ability^43^. For Al_13_@Pt_42_, the effective coordination number (*N*_eff_) is proposed to show the effect of the Al alloying^44^. We carried out a simple linear regression analysis to correlate *E*_ads_(O) and *E*_ads_(OH) adsorbed on T1 and T2 sites with *N*_eff_ of Pt atom, *N*_eff_ = *N*_Pt_ + X*N*_Al_, where subscripts show the corresponding elements, X is the effect coefficient of one Al atom corresponding one Pt atom for *N*_eff_, which is obtained by fitting technique. By using this technique, X = 2.5 is obtained. That is, *N*_eff_ values of Pt_v_ and Pt_e_ on Al_13_@Pt_42_ are 7.5 and 11. The average *N*_eff_ value of Pt_42_ shell consisting of Pt_v_ and Pt_e_ is 10, being larger than 7.4 of Pt_55_ and 9 of Pt(111). The above atomic structural analysis corresponds to the fact that the *d* band center moves towards the lower-energy range from −1.99 eV of Pt_55_ to −2.54 eV of Al_13_@Pt_42_, which effectively illustrates that the presence of Al reduces the adsorption ability of the low-coordinated Pt atoms, as shown in Figure 2(c). On the other hand, the Mulliken charge analysis displayed in Figure 3(a) show that the electrons are transferred from the Al_13_ core to Pt_42_ shell, leading to the formation of negatively charged shell. Therein, the Q(Pt_v_) and Q(Pt_e_) are −0.185 and −0.380*e*, respectively (Q defines as the number of the transferred electrons). After O adsorption on H2 site, Q(O) and Q(Pt_e_) are −0.585 and −0.181*e*, respectively, which means the presence of electrostatic repulsion. However, a completely different situation is found on Pt(111). In Figure 3(b), Pt(111) is nearly electrically neutral with Q(Pt) = −0.006*e*. After O adsorption on fcc site, Q(O) and Q(Pt) are −0.548 and 0.149*e*, respectively. That is, the electrostatic attraction appears for O adsorption on Pt(111). It is plausible that the weaker *E*_ads_(O) of Al_13_@Pt_42_ is just due to this electrostatic repulsion between the electronegative O adatom and the Pt atoms^45^,^46^. In order to demonstrate the effect of the negative charges on *E*_ads_(O), we artificially add electrons Q_add_ on Pt(111) and then calculate the corresponding *E*_ads_(O)^47^. In Figure 3(c), for O adsorption on Pt(111), the Q(Pt) sign is changed from positive to negative and Q(O) is more negative when Q_add_ is increased. Namely, the interaction between O and Pt(111) is changed from electrostatic attraction to electrostatic repulsion. As shown in Figure 3(d), *E*_ads_(O) is weakened as Q_add_ is increased. Furthermore, from the *d*-PDOS of the Pt(111) with different Q_add_ values shown in Figure 3(e), there is little change of the *d* band. Thus, the electrostatic repulsion indeed reduces the *E*_ads_(O). Since OH adsorption has a similar case of O adsorption, we do not show the corresponding results here. In summary, both electronic and atomic structures of Al_13_@Pt_42_ support its high poisoning resistance for ORR.

**Figure 4:**
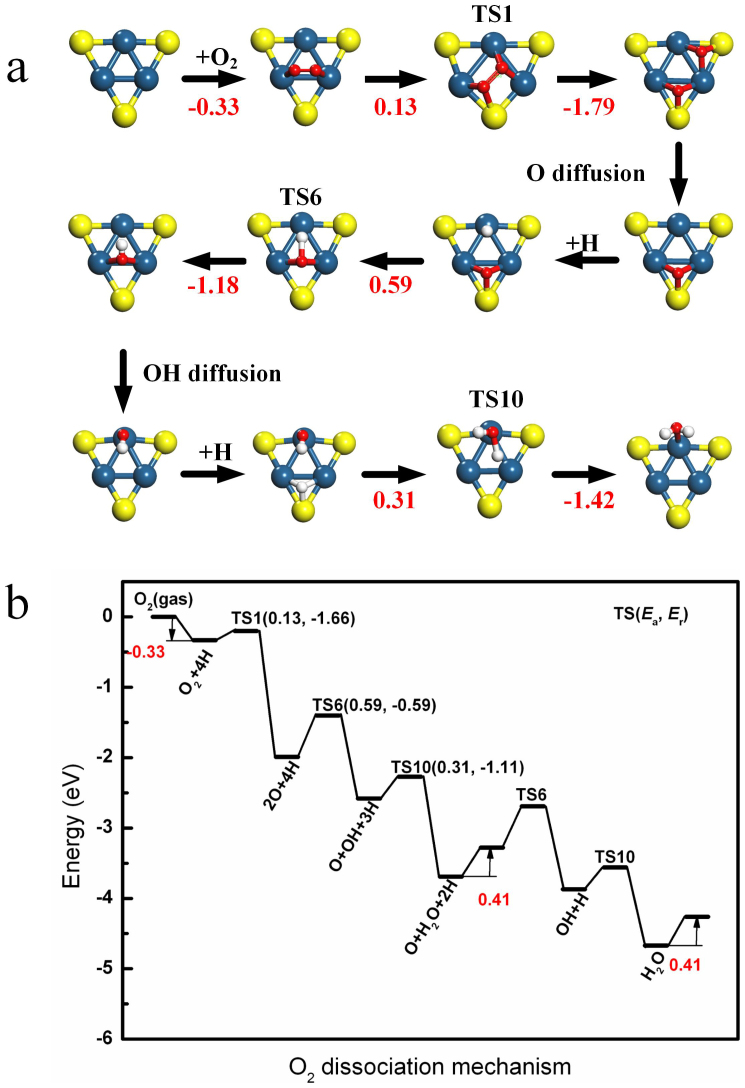
Optimized overall reaction path of O_2_ dissociation mechanism. (a) The structures of initial, transition and final states, respectively. (b) Schematic energy profile.

In order to characterize ORR catalyzed on the Al_13_@Pt_42_, the distinct reaction paths are considered to determine transition states and activation energies or energy barrier (*E*_a_) using nudged elastic band theory (NEB) for all elemental reaction steps involved in ORR in Figure S1 and the corresponding data are listed in Table 3. Firstly, the O_2_ dissociation mechanism, including O_2_ dissociation, OH formation, and H_2_O formation, is considered. The results are shown in Figure 4 and Table 3. For O_2_ dissociation, the *E*_a_ = 0.13 eV. Figure S2 illustrates the spin-polarized partial density of states (PDOS) projected onto the O-O bond where big change in PDOS is present. There is no spin polarization of the adsorbed O_2_ orbitals, and the 5σ, 1π and 2π* orbitals of O_2_ are broadened, which dominate the adsorption of O_2_. It is clear that the partial antibonding orbital 2π* of adsorbed O_2_ are filled compared with that in the gas phase. It is well known that partial population of the antibonding 2π* orbital of O_2_ is responsible for the catalytic activation of the adsorbed O_2_ and stretching of the O-O bond^48^. When O_2_ is adsorbed on Al_13_@Pt_42_ with *E*_ads_(O_2_) = −0.33 eV, the corresponding O-O bond is 1.398 Å (the O-O bond of the gas state is 1.225 Å), due to this charge transfer to the 2π* orbital of O_2_ (0.306 *e*). As results, the O_2_ on Al_13_@Pt_42_ is activated and then dissociated with such a small *E*_a_ value.

In light of Table 3, *E*_a_ = 0.59 eV for OH formation from O and H, which is forbidden on Pt(111) because of the high *E*_a_ of 0.97 eV^18^. It has been demonstrated that the large component of this *E*_a_ comes from O diffusion from hollow site to a bridge site, which is consistent with our results^15^. On Al_13_@Pt_42_, O is easier to diffuse with 0.44 eV diffusion barrier due to the lower *E*_ads_(O) value compared with that of 0.62 or 0.66 eV on Pt(111)^15^,^16^. That is the reason why *E*_a_ value of Al_13_@Pt_42_ is smaller than that of Pt(111) for OH formation from O and H. Thus, the path for OH formation becomes feasible on Al_13_@Pt_42_. For H_2_O formation, *E*_a_ = 0.31 eV, which is comparable with Pt(111)^18^. Similar with Pt(111), the disappearance of the OH diffusion makes *E*_a_ for H_2_O formation lower than that for OH formation^16^. The last step is removal of the adsorbed H_2_O and recovery the surface active site. Once H_2_O is formed, it needs to overcome 0.41 eV for desorption.

## Discussion

Therein, as shown in Figure 4, the rate-limiting step (RDS) of O_2_ dissociation mechanism is located at OH formation from O and H with *E*_a_ = 0.59 eV. On the other hand, for OOH associative mechanism, RDS is located at OOH formation with *E*_a_ = 0.81 eV and *E*_r_ = −0.37 eV. Since *E*_a_ value of RDS of O_2_ dissociation mechanism is lower than that of OOH associative mechanism, the former is more effective. In addition, we have excluded the two-electron reduction to H_2_O_2_ since H_2_O_2_ spontaneous dissociates into OH on Al_13_@Pt_42_, which is consistent with experimental results on Pt and other Pt alloys^49^. When we observe the corresponding data of Pt(111) in Table 3, RDS is located at OH formation from O and H_2_O with *E*_a_ = 0.50 eV^18^,^50^. *E*_a_ value for OH formation on Al_13_@Pt_42_ is comparable with that on Pt(111). It is well known that when *E*_a_ < 0.75 eV, there is room temperature activity^51^. As results, Al_13_@Pt_42_ can effectively catalyze ORR at room temperature.

In summary, the core-shell Al_13_@Pt_42_ cluster is a good ORR candidate for the fuel cell application and possesses at least four superiorities listed below: (1) Excellent cluster stability due to the formation of the Al-Pt covalent bonds; (2) A better activity than Pt(111) due to the optimal O adsorption energy; (3) The maximal Pt atomic utilization of 70% due to the utility of the anti-poisoning edge Pt atoms with consideration of OH adsorption; (4) OH formation with *E*_a_ = 0.59 eV (being comparable with Pt(111) of 0.50 eV) as the RDS with O_2_ dissociation mechanism.

## Methods

Most calculations are performed within the DFT framework as implemented in DMol^3^ code^52^,^53^. The generalized gradient approximation (GGA) with the Perdew–Burke–Ernzerhof (PBE) functional is employed to describe exchange and correlation effects^54^. The All Electron Relativistic (AER) core treat method is implemented for relativistic effects, which explicitly includes all electrons and introduces some relativistic effects into the core^55^. In this work, the double numerical atomic orbital augmented by a polarization *p*-function (DNP) is chosen as the basis set^52^. The orbital cutoff values are 4.5 Å and 4.8 Å for Pt_55_/Pt(111) and Al_13_@Pt_42_, respectively, which lead to the difference of the atomic energies is within 0.1 eV atom^−1^, compared with the cutoff value of 6.5 Å. Thus, the value satisfies the accuracy required. A smearing of 0.005 Ha (1 Ha = 27.21 eV) to the orbital occupation is applied to achieve accurate electronic convergence. The spin-unrestricted method is used for all calculations. The convergence tolerance of energy is 1.0 × 10^−5^ Ha, maximum force is 0.002 Ha/Å, and maximum displacement is 0.005 Å in Dmol^3^. Note that the DNP basis set is the most accurate for our studied systems in Dmol^3^ code when Pt element is included in any considered system and is comparable to the Gaussian 6–31(d) basis^56^ while DNP results have shown excellent consistency with experiments in literatures^57^.

It is known that the cell size effect for the calculations of 38 atomic clusters is negligible when the size is large than 25 Å^58^. In our case, we consider the calculations of 55 atomic clusters. Thus, we have tested cubic boxes with sizes of 25 Å and 30 Å. The results are shown in Table S1. It is found from Table S1 that the results with sizes of 25 Å and 30 Å are consistent each other within energy difference smaller than 0.001 Ha. Thus, the box size of 25 × 25 × 25 Å^3^ is chosen for our system.

A three-layer *p*(3 × 3) periodic slab is taken to simulate Pt(111) surface where the two bottom layers are fixed. To confirm the reliability of this Pt(111) model, the *E*_ads_(O) and *E*_ads_(OH) values on a four-layer slab with two fixed layers and a five-layer slab with three fixed layers are calculated and listed in Table S2. It is shown that compared with the three-layer slab, the *E*_ads_(O) and *E*_ads_(OH) values on the two cases have errors of 0.03, which do not change all results what we have obtained. Therefore, our Pt(111) model has reasonable accuracy. The k-points are Gamma point for clusters and (2 × 2 × 1) for Pt(111), respectively. The minimum energy paths (MEPs) for ORR are obtained by LST/QST tools in DMol^3^ code.

A conductor-like screening model (COSMO) is used to simulate a H_2_O solvent environment throughout the whole process in DMol^3^ code^59^ where the dielectric constant is set as 78.54 for H_2_O solvent. It is noteworthy that this environment is necessary to describe the solvation^50^. To confirm this consideration, the adsorption energy values of O_2_, O, H_2_O and OH in gas and solvent environments are calculated and shown in Table S3. As shown in the table, there are evident differences in the both cases. For O_2_, O and H_2_O adsorption under solvent environment, *E*_ads_ are stronger while *E*_ads_(OH) is weaker, compared with gas environment.

It is well known that there is the convergence failure of magnetic systems in DMol^3^ code. To compare the stability among the different M_13_@Pt_42_ clusters, the core-shell interaction energy *E*_cs_ and the dissolution potentials of Pt_42_ shell *U*_diss_(M_13_@Pt_42_) are calculated in CASTEP code with ultrasoft pseudopotentials^60^. The PBE is employed to describe exchange and correlation effects^54^. The use of a plane-wave kinetic energy cutoff of 400 eV is shown to give excellent convergence of total energies. The convergence tolerance of energy is 1.0 × 10^−5^ eV/atom, maximum force is 0.05 eV/Å, and maximum displacement is 0.005 Å in CASTEP. The 0.2 eV smearing is adopted for calculations.

To analyze the structural stability of alloy clusters with different numbers of Al atoms, the average binding energy of the cluster *E*_b_ is adopted, 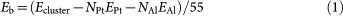
where *E*_cluster_, *E*_Pt_ and *E*_Al_ are the total energies of Pt_55_ or Al_13_@Pt_42_ clusters, Pt atom, and Al atom, respectively. *N*_Pt_ and *N*_Al_ denote the numbers of Pt and Al atoms.

The core-shell interaction energy *E*_cs_ is calculated as following, 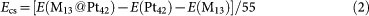
where E(M_13_@Pt_42_), *E*(Pt_42_) and *E*(M_13_) are the total energies of M_13_@Pt_42_ clusters, Pt_42_ shell and M_13_ core, respectively.

Following the idea of Noh et al.^26^, we define the dissolution potential of M_13_@Pt_42_ cluster as the lowest potential at which the Pt-skin layer dissolves into acidic solution. Specifically, we considered the electrochemical reaction of M_13_@Pt_42_ cluster of eq. (3), 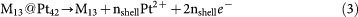
where n_shell_ is the number of Pt atoms in the M_13_@Pt_42_ (n_shell_ = 42). The dissolution potential of the Pt_42_ shell is calculated by, 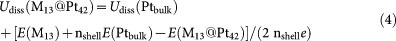
where *U*_diss_(M_13_@Pt_42_) and *U*_diss_(Pt_bulk_) are the dissolution potentials of the outmost shell of M_13_@Pt_42_ clusters and that of a bulk Pt, respectively. *E*(Pt_bulk_) denotes the total energy of bulk Pt. Here, *U*_diss_(Pt_bulk_) = 1.188 V.

The adsorption energies (*E*_ads_) of adsorbates on these clusters are calculated through, 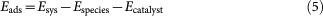
where *E*_species_, *E*_catalyst_ and *E*_sys_ are the total energy of an isolated adsorbate species, the catalyst [Pt(111), Pt_55_ and Al_13_@Pt_42_] and the adsorption system, respectively. *E*_ads_ < 0 corresponds to an exothermic adsorption process.

## Supplementary Material

Supplementary Information Supporting information for Al13@Pt42 Core-Shell Cluster for Oxygen Reduction Reaction

## Figures and Tables

**Figure 1:**
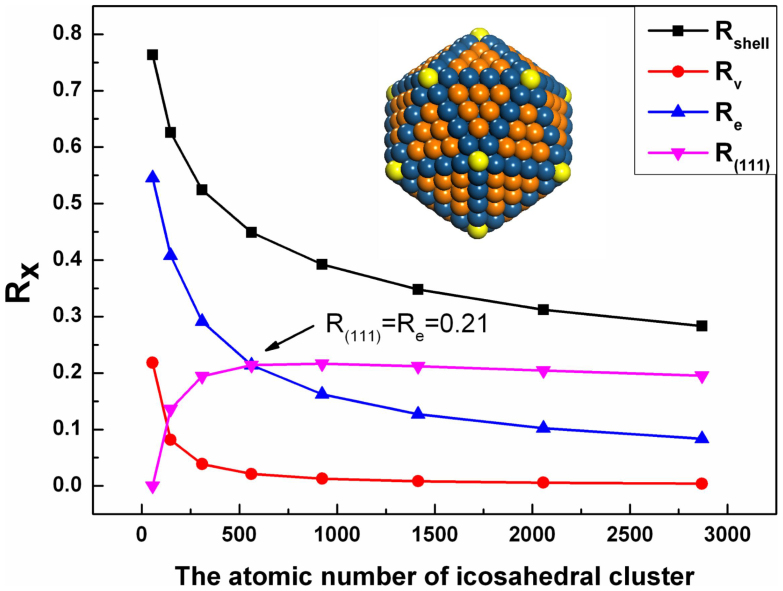
Size-dependent percentage of atoms on (111), edge and vertex, expressed by R_(111)_, R_e_ and R_v_. R_shell_ denotes the percentage of atoms on the shell, and R_shell_ = R_(111)_ + R_e_ + R_v_. Atoms in yellow, blue and orange denote Pt_v_, Pt_e_ and Pt_(111)_atoms. Inset is the structure of Pt_561_ where R_e_ = R_(111)_.

**Table 1:** The calculated core-shell interaction energies *E*_cs_ and the Pt_42_ shell dissolution potentials *U*_diss_(M_13_@Pt_42_). The units are eV/atom and V, respectively

	Pt_55_	Al_13_@Pt_42_	Fe_13_@Pt_42_	Co_13_@Pt_42_	Ni_13_@Pt_42_
*E*_cs_	−0.52	−0.84	−0.75	−0.62	−0.53
*U*_diss_(M_13_@Pt_42_)	0.816	1.101	1.087	0.995	0.933

**Table 2:** The calculated adsorption energy values of *E*_ads_(O) and *E*_ads_(OH) on Pt_55_, Al_13_@Pt_42_, and Pt(111) (The adsorption sites are described in caption of Figure 2b).The values in parentheses show differences between *E*_ads_(O) and *E*_ads_(OH) values on Al_13_@Pt_42_ and that on Pt(111)

	*E*_ads_(O)		*E*_ads_(OH)	
	H1	H2	T1	T2
Pt_55_	−4.56(−0.05)	−4.71(−0.20)	−3.13(−0.68)	−2.91(−0.46)
Al_13_@Pt_42_	−4.15(0.36)	−4.25(0.26)	−2.81(−0.36)	−2.15(0.30)
Pt(111)	−4.51		−2.45	

**Table 3:** The preferred activation energies (*E*_a_) and reaction energies (*E*_r_) for elemental steps in ORR. All results are in unit of eV

	Al_13_@Pt_42_		Pt(111)	
Reaction steps	*E*_a_	*E*_r_	*E*_a_	*E*_r_
O_2_ → 2O	0.13	−1.66	0.00	−2.18
O + H → OH	0.59	−0.59	0.97	−0.07
OH + H → H_2_O	0.31	−1.11	0.24	−0.56
H_2_O → H_2_O(gas)		0.41		0.58
O_2_ + H → OOH	0.81	−0.37	0.22	−0.19
OOH → O + OH	0.28	−0.91	0.00	−2.07
O + H_2_O → 2OH	0.57	0.57	0.50	0.49
O(H1) → O(H2)	0.44		0.62–0.66	

^a^The O diffusion energy barrier on Pt(111) are from references [15,16], the H_2_O desorption barrier is from reference [50]while other data on Pt(111) come from reference [18]
