# Supplementary material for: Al13@Pt42 Core-Shell Cluster for Oxygen Reduction Reaction
Source: Sci Rep. 2014 Jun 6;4:5205. doi: 10.1038/srep05205 (PMC5381497; doi:10.1038/srep05205)
Supplement: Supplementary Information — Supporting information for Al13@Pt42 Core-Shell Cluster for Oxygen Reduction Reaction [file srep05205-s1.pdf]

Supporting Information

for

$Al_{13}@Pt_{42}$ Core-Shell Cluster for Oxygen Reduction Reaction

B. B. Xiao, Y. F. Zhu,* X. Y. Lang, Z. Wen, Q. Jiang*

Key Laboratory of Automobile Materials, Ministry of Education, and Department of Materials Science and Engineering, Jilin University, Changchun 130022, China

Figure S1. Reaction energy path for elemental steps of ORR on $Al_{13}@Pt_{42}$. TS1 and TS2 are the transition states (TS) of O_2 dissociation ($O_2 \rightarrow 2O$); TS3 and TS4 denote TS of OOH formation (O_2+H→OOH); TS5 is TS of OOH dissociation (OOH→O+OH); TS6 and TS7 are TS of OH formation from the reactants O and H (O+H→OH) while TS8 is TS of OH formation from O and H_2O (O+H_2O→2OH); TS9 and TS10 show the transition states of H_2O formation (OH+H→H_2O). For O_2 adsorption, the corresponding O-O bond length and the electron number are shown. In addition, the coadsorption energy E_{ads}(H+O) and E_{ads}(OH+H) are given.

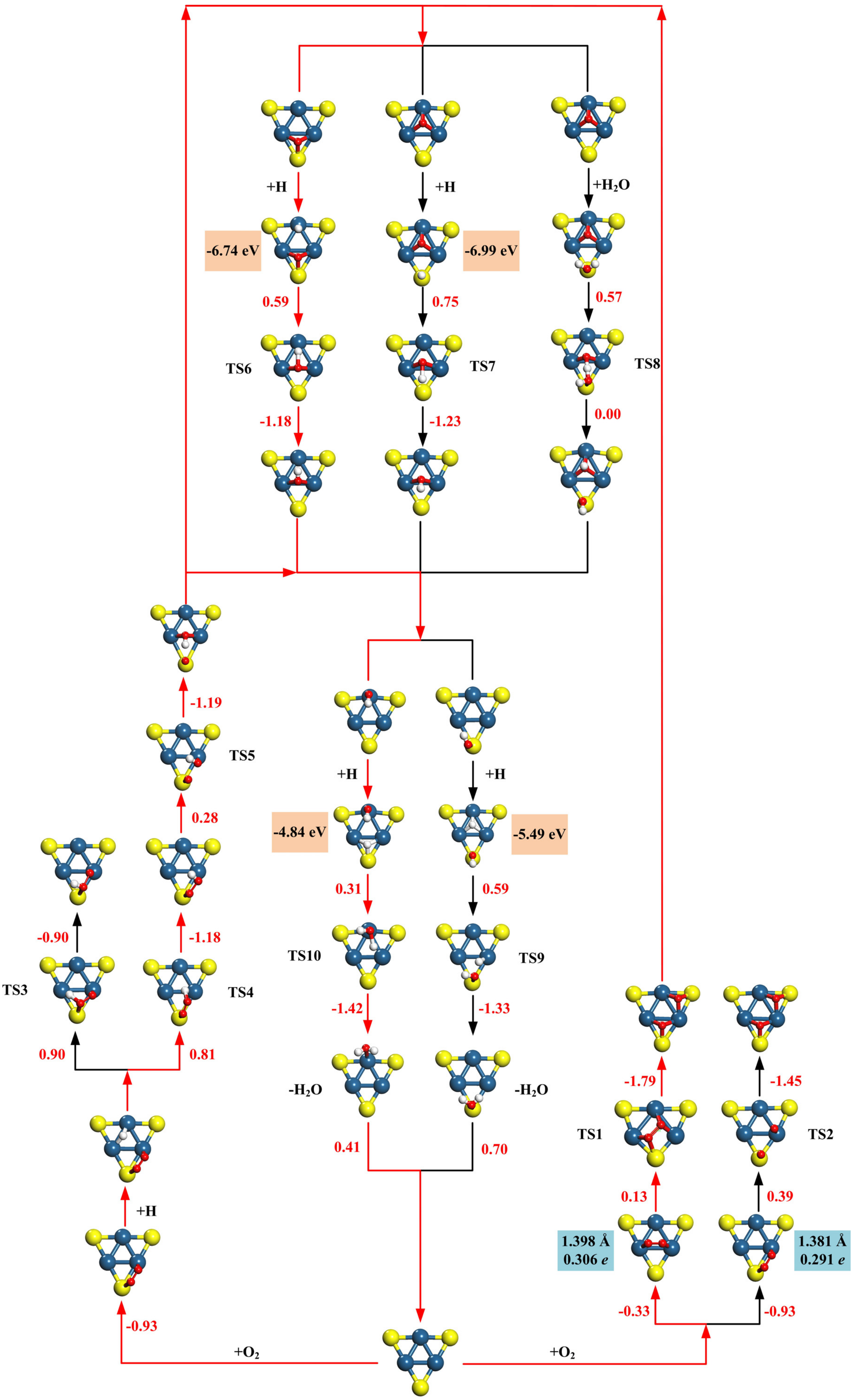

+H
+H
$+H_2O$
-6.74 eV
-6.99 eV
0.59
0.75
0.57
TS6
TS7
TS8
-1.18
-1.23
0.00
-1.19
TS5
+H
+H
0.28
-4.84 eV
-5.49 eV
0.31
0.59
-0.90
-1.18
TS10
TS9
TS3
TS4
-1.42
-1.33
0.90
0.81
$-H_2O$
$-H_2O$
-1.79
-1.45
0.41
0.70
TS1
TS2
+H
0.13
0.39
1.398 Å
0.306 *e*
1.381 Å
0.291 *e*
-0.93
-0.33
-0.93
$+O_2$
$+O_2$

Figure S2. Partial density of states (PDOS) for O_2 adsorbed on bridge site formed by Pt_e atoms. Insert is the enlarged PDOS near the Fermi level.

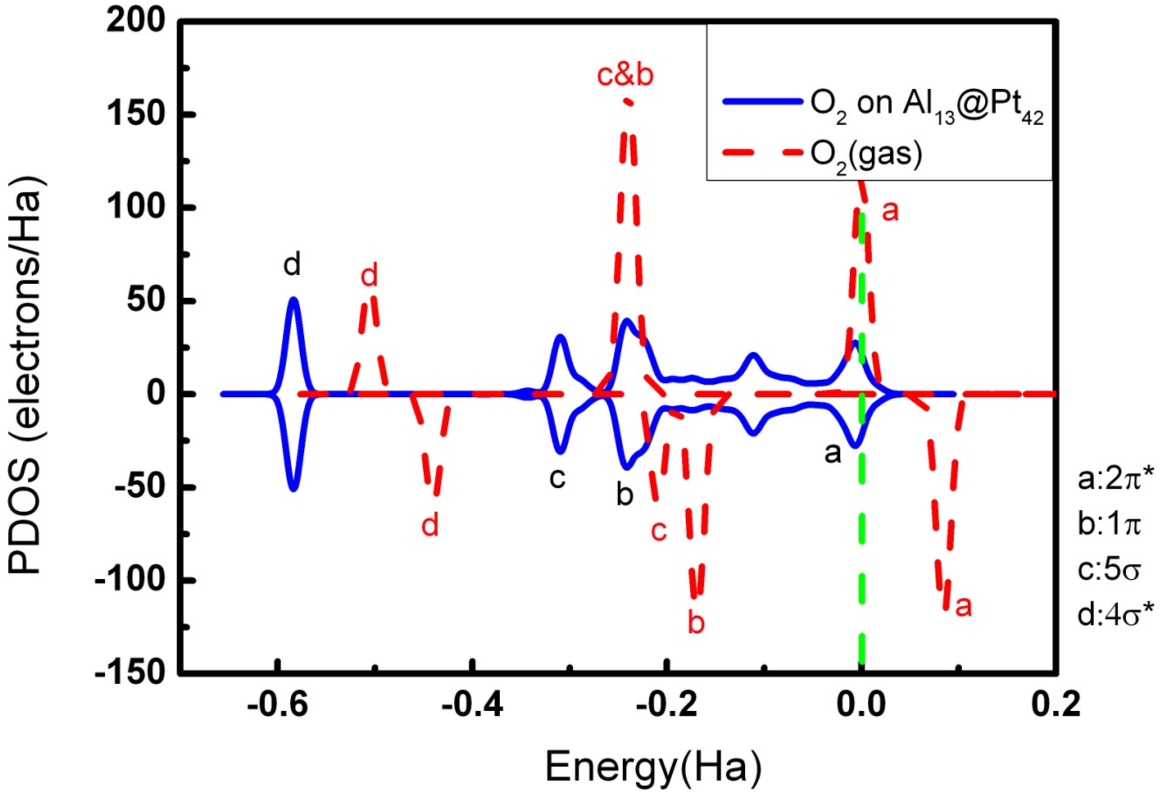

Table S1. The convergence test of cell sizes. The total energy of $Al_{13}@Pt_{42}$ cluster and two adsorption systems (O adsorption on H2 site and OH adsorption on T2 site) in three different cell sizes (25 × 25 × 25 $Å^3$ and 30 × 30 × 30 $Å^3$) are listed. All results are in unit of Ha.

	25 × 25× 25	30 × 30 × 30
$Al_{13}@Pt_{42}$	-633082.2295226	-633082.2291524
O on H2 site	-633157.5480120	-633157.5474880
OH on T2 site	-633158.1764345	-633158.1758359

Table S2. The adsorption energies E_{ads} of Pt(111) with different layers in eV. Pt(111)-n-m denotes that the slab is modeled by n layers with the bottom m layers fixed.

	Pt(111)-3-2	Pt(111)-4-2	Pt(111)-5-3
O on fcc site	-4.51	-4.39	-4.38
OH on atop	-2.45	-2.37	-2.39

Table S3. The adsorption energies in a gas phase environment ($E_{ads\text{-}gas}$) and in a H_2O solvent environment (E_{ads}) of $Al_{13}@Pt_{42}$. The solvation energies E_{solv} of ORR intermediates defined as $E_{solv} = E_{ads} – E_{ads\text{-}gas}$. All results are in unit of eV. B1 is the bridge formed by Pt_v and Pt_e atoms while B2 is formed by only Pt_e atoms.

	$E_{ads}(O_2)$		$E_{ads}(O)$		$E_{ads}(OH)$		$E_{ads}(H_2O)$	
	B1	B2	H1	H2	T1	T2	T1	T2
$E_{ads\text{-}gas}$	-0.89	-0.29	-4.13	-4.22	-2.87	-2.21	-0.61	-0.39
E_{ads}	-0.93	-0.33	-4.15	-4.25	-2.81	-2.15	-0.70	-0.41
E_{solv}	-0.04	-0.04	-0.02	-0.03	0.06	0.06	-0.09	-0.02
